# Cognitive changes are associated with increased blood-brain barrier leakage in non-brain metastases lung cancer patients

**DOI:** 10.1007/s11682-022-00745-3

**Published:** 2022-11-22

**Authors:** Da-Fu Zhang, Zhen-Hui Li, Zhi-Ping Zhang, Yin-Fu He, Bin-Li Shang, Xiu-Feng Xu, Ying-Ying Ding, Yu-Qi Cheng

**Affiliations:** 1 Department of Psychiatry, the First Affiliated Hospital of Kunming Medical University, No. 295 Xichang Road, Kunming, 650032 Yunnan China; 2 Department of Radiology, the Third Affiliated Hospital of Kunming Medical University, Yunnan Cancer Hospital, Yunnan Cancer Center, No. 519 Kunzhou Road, Kunming, 650118 Yunnan China; 3 Yunnan Clinical Research Center for Mental Disorders, Kunming, 650032 Yunnan China

**Keywords:** Lung cancer, Staging, Cognitive impairment, Neuroimaging, Blood–brain barrier

## Abstract

To explore the relationship between cognitive function and blood–brain barrier leakage in non-brain metastasis lung cancer and healthy controls. 75 lung cancers without brain metastasis and 29 healthy controls matched with age, sex, and education were evaluated by cognitive assessment, and the Patlak pharmacokinetic model was used to calculate the average leakage in each brain region according to the automated anatomical labeling atlas. After that, the relationships between cognitive and blood–brain barrier leakage were evaluated. Compared with healthy controls, the leakage of bilateral temporal gyrus and whole brain gyrus were higher in patients with lung cancers (*P* < 0.05), mainly in patients with advanced lung cancer (*P* < 0.05), but not in patients with early lung cancer (*P* > 0.05). The cognitive impairment of advanced lung cancers was mainly reflected in the damage of visuospatial/executive, and delayed recall. The left temporal gyrus with increased blood–brain barrier leakage showed negative correlations with delayed recall (r = -0.201, P = 0.042). An increase in blood–brain barrier leakage was found in non-brain metastases advanced lung cancers that corresponded to decreased delayed recall. With progression in lung cancer staging, blood–brain barrier shows higher leakage and may lead to brain metastases and lower cognitive development.

**Supplementary Information:**

The online version contains supplementary material available at 10.1007/s11682-022-00745-3.

## Introduction

Lung cancer (LC) is the most common cancer and the leading cause of cancer death in both men and women(Feng et al., 2019; Sung et al., 2021). Due to untimely diagnosis, most lung cancers are advanced at the time of diagnosis(Alberg et al., 2013). The incidence of brain metastases (BMs) ranges from 22 to 54% and can occur at different stages of tumor development, especially in patients with advanced disease(Alberg et al., 2005). BM is present in 10% of patients at the time of initial diagnosis(Villano et al., 2015). The brain is the only site of tumor recurrence in 50% of patients(Hu et al., 2006). Many chemotherapeutic agents are relatively poor penetrators of the central nervous system (CNS), allowing tumor cells to survive and develop into BM in the CNS. Although recent therapeutic advances including intrathecal chemotherapy, molecular targeted therapies, and immunotherapy have somewhat prolonged the survival time of BM patients, the prognosis of BM remains poor with a median overall survival of 7–13 months(Kondziolka et al., 2005). BM may lead to different focal neurological symptoms and cognitive dysfunction(Chen et al., 2007; Noh & Walbert, 2018).

The blood–brain barrier (BBB) is a selective "gatekeeper" composed of endothelial cells, pericytes, and astrocyte end-foot. BBB separates the brain from systemic circulation and protects it from foreign infectious and/or toxic agents(Arvanitis et al., 2020; Cheng & Perez-Soler, 2018; Fares et al., 2020b). It is estimated that BBB blocks the transport of about 98% of the molecules in systemic circulation(Pardridge, 2005). The integrity of the BBB is essential to block the entry of most tumor cells.

The pathogenesis of BM is complex, and BBB dysfunction is one of its pathogenic mechanisms. Metastasis of lung cancer cells to the brain requires cancer cells to invade the circulatory system from the primary site and colonize in brain parenchyma through the invasion of BBB(Fares et al., 2020a, 2020b). Research has shown that BBB is damaged in this process, resulting in increasing permeability of BBB(Langley & Fidler, 2013). BBB dysfunction (BBBD) is also a feature of other brain diseases, such as stroke(Serlin et al., 2019), subarachnoid hemorrhage (Lublinsky et al., 2019), and epilepsy (Rüber et al., 2018). Recently considered as a potential biomarker for predicting outcome(Bar-Klein et al., 2017; Kamintsky et al., 2020; Lublinsky et al., 2019; Serlin et al., 2019). Although the detailed mechanism of BBBD is unknown, paracellular leakage due to dysfunction or downregulation of tight junction proteins, as well as enhanced endothelial barrier transport across cells, are considered to be pathogenic factors(Andreone et al., 2017; Zhang et al., 2017). Currently, there is no clinically standardized method to objectively assess changes in microvascular permeability in vivo.

Dynamic enhanced magnetic resonance imaging (DCE-MRI) is increasingly being used to assess cerebrovascular permeability in neurological diseases(Sweeney et al., 2019). The widely used DCE-MRI approach is based on extended Tofts or Patlak models(Patlak et al., 1983; Sourbron & Buckley, 2013; Tofts & Kermode, 1991). In lung cancer patients without brain metastasis, there is a lack of quantitative data on BBB leakage and a lack of understanding of the spatial scope of BBB. Therefore, we aim to quantify the BBB leakage in lung cancer patients without brain metastasis and to examine the relationship between BBB leakage and cognitive function.

## Materials and methods

### Participant

This study was approved by the Ethics Committee of the third affiliated Hospital of Kunming Medical University (NO. SLKYLX202118). all subjects signed informed consent before participating in the research. This work was conducted by the principles of the Declaration of Helsinki and its later amendments.

**Fig. 1:**
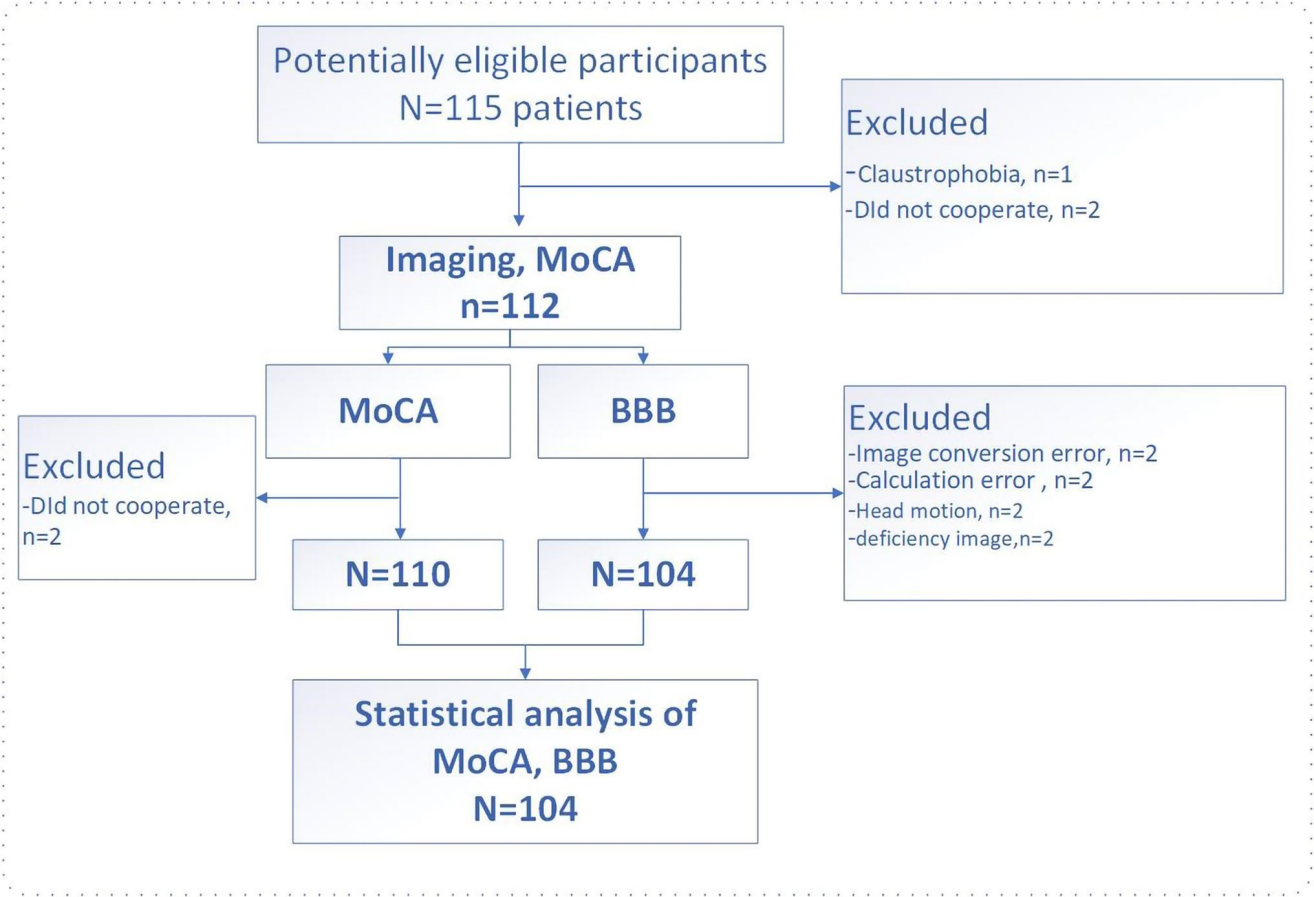
Flow chart of subjects enrollment

This is a cross-sectional study of lung cancer patients, which plans to scan untreated patients and include age/sex-matched healthy controls (HCs). From August 2021 to February 2022, 75 lung cancer patients (39 patients with early-stage lung cancer and 36 patients with advanced lung cancer) from the Department of Thoracic Surgery, Third Affiliated Hospital of Kunming Medical University, and 29 HCs (enrolled through online advertisement, matched for age and gender) participated in this study. All participants were untreated (i.e., surgery, radiotherapy, or immunotherapy) before pathological confirmation. According to TNM (tumor, node, metastasis) staging (8th edition) (Detterbeck et al., 2017), early-stage patients are those with stage I, advanced-stage patients are those with stage II to IV. Some participants were excluded due to excessive head movement during scanning, contrast allergy, and calculation failure (Fig. 1). Exclusion criteria for all participants were: receipt of prophylactic cranial irradiation; the presence of brain metastases; known history of stroke, cranial trauma, epilepsy, Alzheimer's disease, Parkinson's disease, other acute psychiatric or neurological disorders; history of major medical illness (e.g., anemia, severe heart disease, thyroid dysfunction, or abnormal liver or kidney function); and severe vision or hearing loss.

### MRI acquisition

All data were acquired on a 3.0 T MRI scanner (Discovery MR750, GE Healthcare, Waukesha, Wisconsin, USA) with a 21-channel receiver array head and neck coil, acquired in parallel. Tight but comfortable foam pads are used to minimize head movement; earplugs and headphones are used to reduce scanner noise. For each participant, routine MRI sequences, including T2 and T1 weighted imaging and T2 fluid-attenuated inversion recovery (FLAIR) imaging, were performed to ensure that there were no visible brain lesions or brain metastases.

The sequences for BBB assessment included the following steps: (1) a T1 -weighted 3D axial sequence with variable flip angles (3D-SPGR, TR 5.9 ms, TE 2.0 ms, flip angle 5° and 14°, FOV 24 × 20.4, acquisition matrix 256 × 180, slice thickness 4. 0 mm, interval 0, bandwidth 62.5 kHz); and (3) a T1-weighted 3D axial dynamic scan (LAVA, TR 5.9 ms, TE 2.0 ms, flip angle 14°, FOV 24 × 20.4, acquisition matrix 256 × 180, slice thickness 4.0 mm, interval 0, bandwidth 62.5 kHz) acquired within 650 s after intravenous injection of the magnetic contrast gadolinium (0.05 mmol/kg, flow rate 3.0 mL/s).

### BBB data preprocessing

Because the contrast medium leakage caused by the blood–brain barrier leakage increases the T1-weighted signal in the tissue, the contrast medium leakage can be calculated to reflect the BBB leakage. To achieve this, SPM12 (University College London, www.fil.ion.ucl.ac.UK/spm) is first used to preprocess data. The specific steps include: image format conversion; head movement correction; image registration; image segmentation; standardization; smoothing; individual space conversion.

**Fig. 2:**
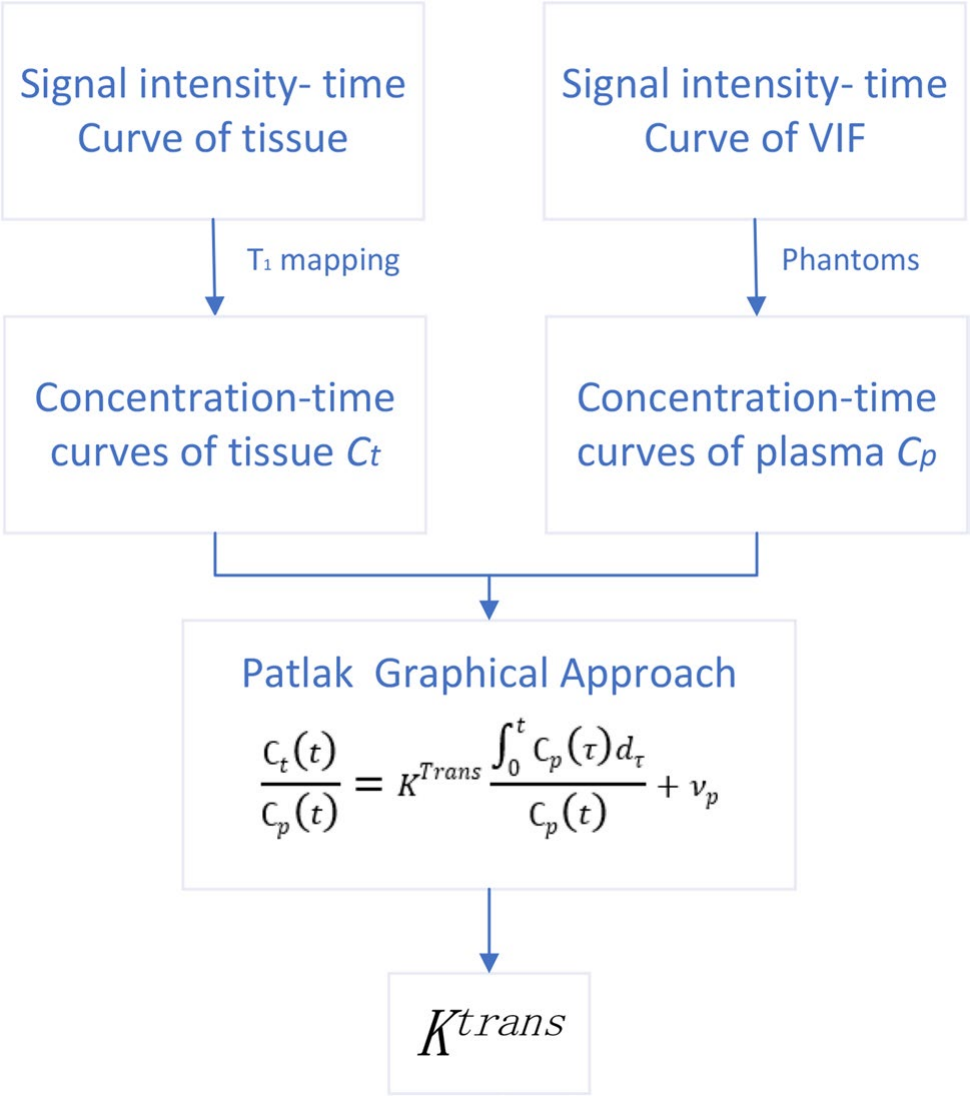
Converts the time-signal strength curve of tissue and vascular input function (VIF) into a time-concentration curve. The leakage rate (K^trans^) was calculated by Patlak graphic method

Previous studies have shown that patlak model is the more accurate than other models in diseases with slight BBB damage(Barnes et al., 2016; Heye et al., 2016; Patlak et al., 1983). And using DCE-MRI to measure the subtle leakage of the BBB has moderate to excellent repeatability(Wong et al., 2017). Therefore, the patlak model will be used to calculate the K^trans^ value in this study. The Patlak method uses a two-compartment model, in which it is assumed that there is no reflux and infinite flow, so the leakage rate is approximately the product of vascular permeability (P) and the surface area (S) per unit tissue mass. For the Patlak graphic method, the two main determinants of the accuracy of measuring BBB are the estimation of blood concentration curve based on T1 signal intensity and the determination of vascular input function (VIF)(Larsson et al., 2009) (Fig. 2). The common method of T1 mapping is to change the flip angle(Brookes et al., 1999). The determination step of VIF also plays a key role in the estimation of kinetic parameters. The VIF is calculated by selecting the region of interest (ROI) of the superior sagittal sinus(Lavini & Verhoeff, 2010). After that, the Patlak model is used to calculate the volume transfer constant K^trans^ (minite^−1^) based on MATLAB (R2017b). The K^trans^ value is used to reflect the leakage of BBB.

K^trans^ is calculated as voxels. Based on the AAL atlas(Tzourio-Mazoyer et al., 2002).

It is assumed that the increased BBB leakage in the brain regions related to cognitive function, such as frontal gyrus, parietal gyrus, temporal gyrus, hippocampus, and cingulate gyrus(Correa et al., 2017; de Ruiter et al., 2012; Inagaki et al., 2007; Mentzelopoulos et al., 2021; Shiroishi et al., 2017; Simó et al., 2015), leads to the decrease of cognitive function. therefore, we use these brain regions to extract the average K^trans^ value of BBB leakage for ROI for analysis.

## Statistical analyses

The continuous variables were compared by ANOVA or Kruskal–Wallis test, and the classified variable ratio was compared by the χ ^2^ test. All statistical analyses are carried out using the software R (version 3.6.3, http://www.r-project.org/). The R language and GraphPad Prism (9.0.0, GraphPad Software, Inc., San Diego, California) were used for visualization. Multiple comparisons are corrected by Bonferroni.

## Result

### Demographic and clinical features

A summary of all detailed demographic data and histological diagnosis and tumor staging is shown in Table 1. There was no significant difference in sex, age, smoking, and KPS score between LCs and HCs (P > 0.05). The longest diameter of the tumor in the eLC group was smaller than that in the aLC group (P < 0.001). The tumor markers CEA, NSE, CYFRA21-1 and SCC in the aLC group were higher than those in the eLC group.

**Table 1:** Demographic and clinical features of patients with Lung cancer

	Controls(n = 29)	Early lung cancer (n = 39)	Advanced lung cancer (n = 36)	*χ2/F/μ*	pvalue
gender					
male/female	15/14	15/24	24/12	5.986	0.05^a^
Age(mean ± SD), year	51.07 ± 9.67	55.59 ± 8.23	56.06 ± 8.99	3.059	0.051^b^
Smoking(%)	10/29	9/39	16/36	3.84	0.147^a^
Tumor diameter (cm) (mean ± SD)		1.62 ± 0.94	4.93 ± 2.35	-8.103	0.000^*c^
KPS score(mean ± SD)	95.00 ± 7.77	96.15 ± 7.11	94.44 ± 6.95	0.568	0.568^b^
Years of education (mean ± SD)	10.00 ± 3.67	8.95 ± 3.50	7.92 ± 4.89	F = 2.102	0.127
Pathological type				χ2	
Squamous cell carcinoma		7	13	7.396	0.092^a^
Adenocarcinoma		29	18		
Small Cell Lung Cancer		3	5		
Tumor markers (25%,50%,75%)				Z	
CEA		1.53, 2.25, 3.57	1.87,4.38, 10.33	446.50	0.007^*d^
NSE		9.60, 11.80, 13.40	11.75, 13.70,21.96	397.50	0.001^*d^
CYFRA21-1		1,40, 2.00,2.60	2.625,4.500,7.025	223.00	0.000^*d^
SCC		0.70,0.80,1.00	0.72,1.10,1.47	493.00	0.026^*d^

Data are expressed as ‾x ± s, n (%) or Interquartile Range (P_25_,P_50_,P_75_). ^a^The P values are obtained by using the χ2 test. ^b^The P values are obtained by using one-way ANOVA. ^c^The P values are obtained by using two-sample t-test. ^d^The P values are obtained by the Kruskal–Wallis test. ^*^P < 0.05 is considered significant. CEA, carcinoembryonic antigen. NSE, neuron-specific enolase. CYFRA21-1, cytokeratins21-1. SCC, squamous cell carcinoma antigen

### The BBB leakage in patients with lung cancer at different stages

**Fig. 3:**
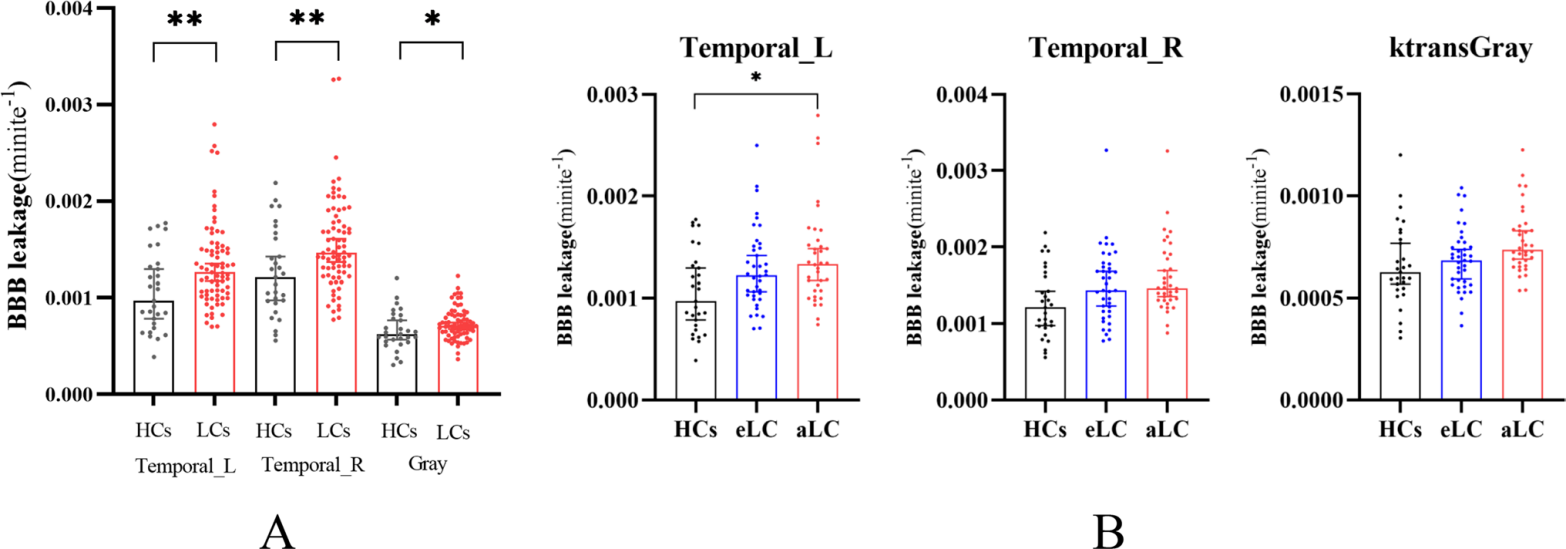
Comparison of BBB leakage between patients with LC HCs. A Comparison of LCs and HCs. B Comparison of eLCs, aLCs and HCs gyrus

**Fig. 4:**
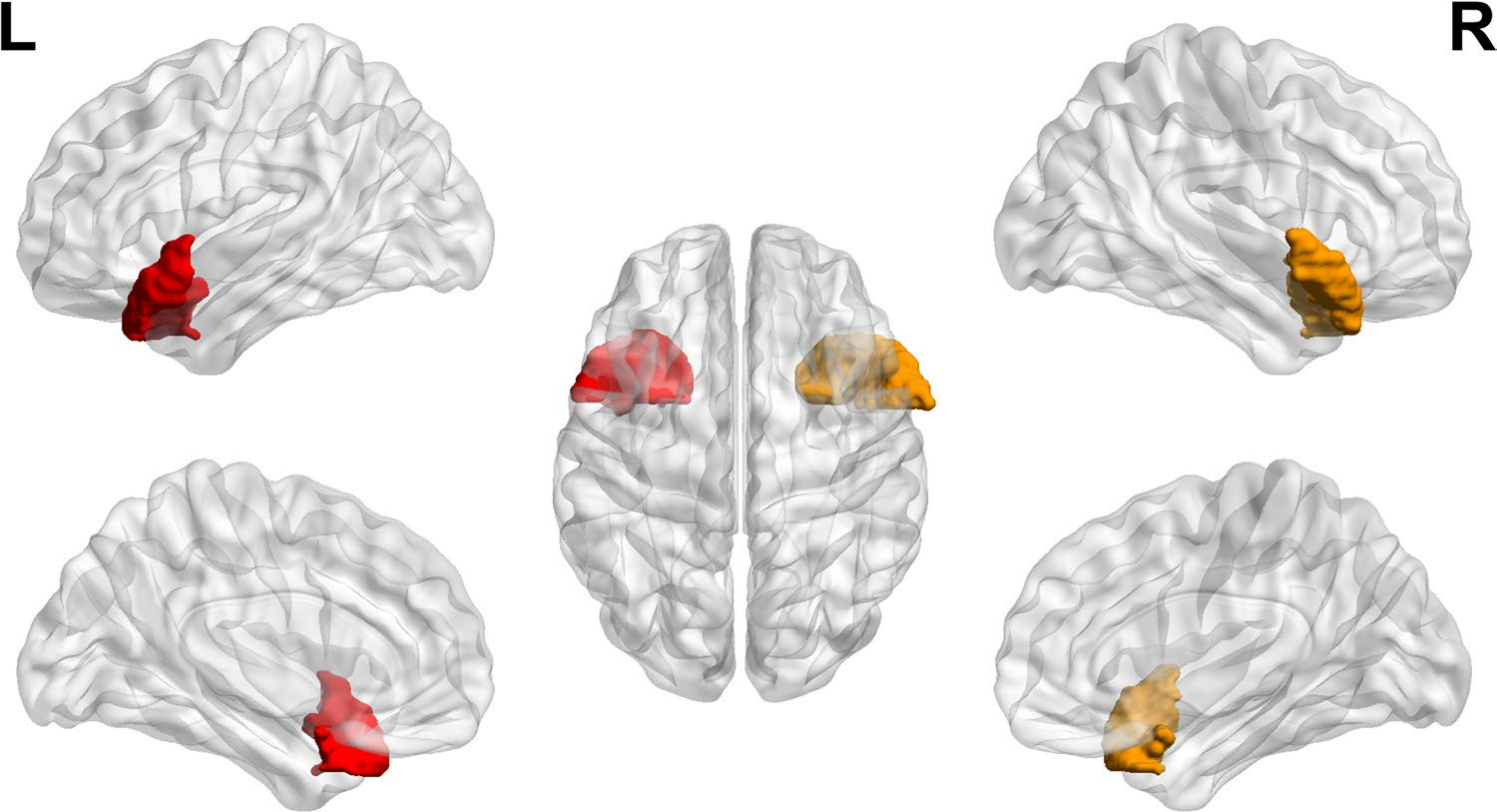
Location and distribution of hyperosmotic brain areas in the brain

We extracted and analyzed the average K^trans^ values of BBB in bilateral frontal gyrus, parietal gyrus, temporal gyrus, hippocampus and cingulate gyrus. Compared with HCs, the K^trans^ of bilateral temporal gyrus and whole brain gyrus were increased in LCs. mainly in patients with advanced lung cancer (*P* < 0.05), but not in patients with early lung cancer (*P* < 0.05). As shown in Figs. 3 and 4.

### Cognitive impairment in patients with advanced lung cancer

**Fig. 5:**
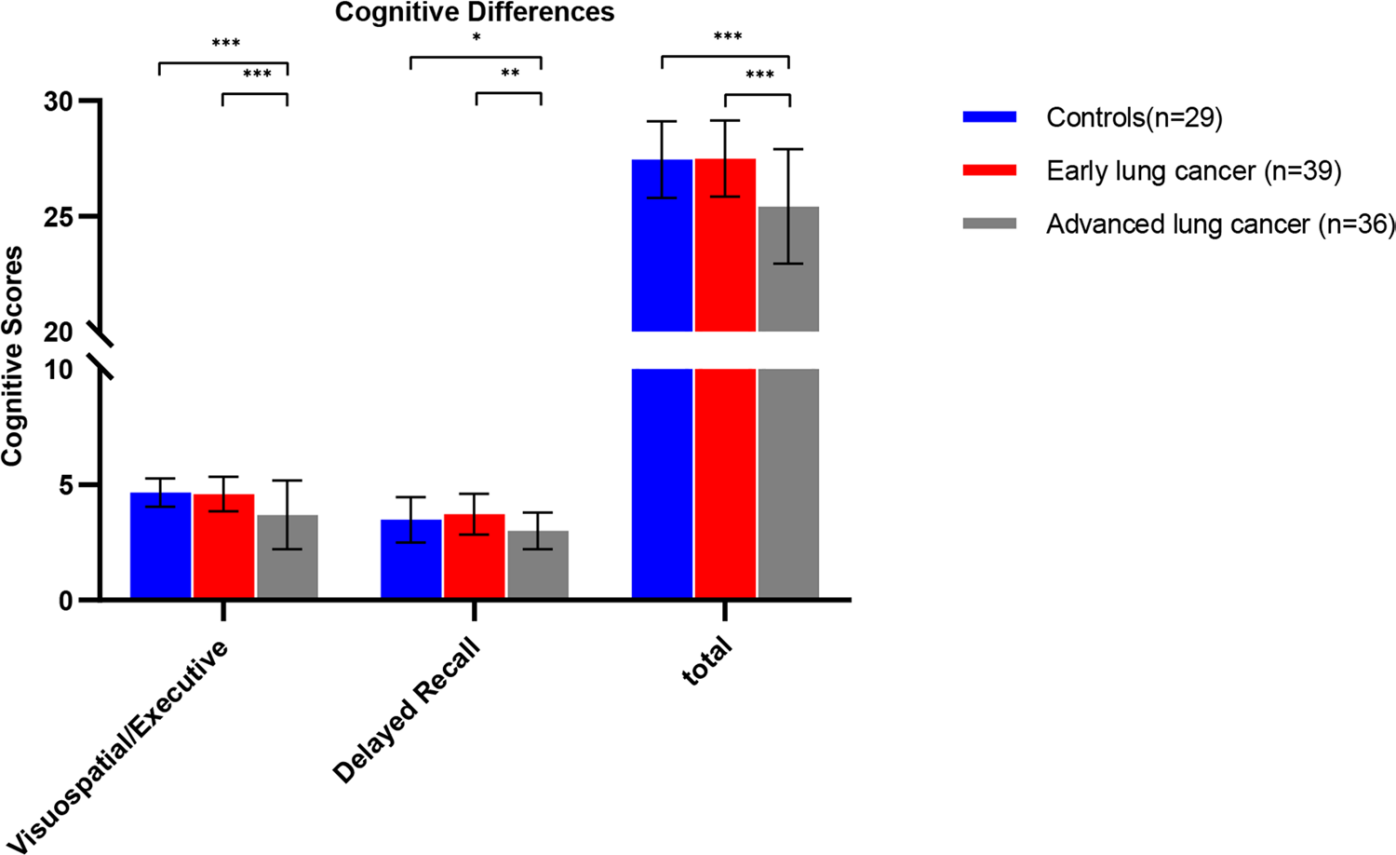
Difference in cognitive function between patients with lung cancer in different stages and HCs. Total = total score of cognitive function

The cognitive impairment of aLCs was mainly reflected in the dysfunction of visuospatial/executive, and delayed recall. There was no significant difference between eLCs and HCs. and it was considered that the cognitive function of eLCs had not been impaired. Moreover, the damage of advanced lung cancer is more obvious than that of early patients. As detailed in Fig. 5.

### Correlation analysis between increased brain leakage and cognitive function in patients with lung cancer at different stages

**Fig. 6:**
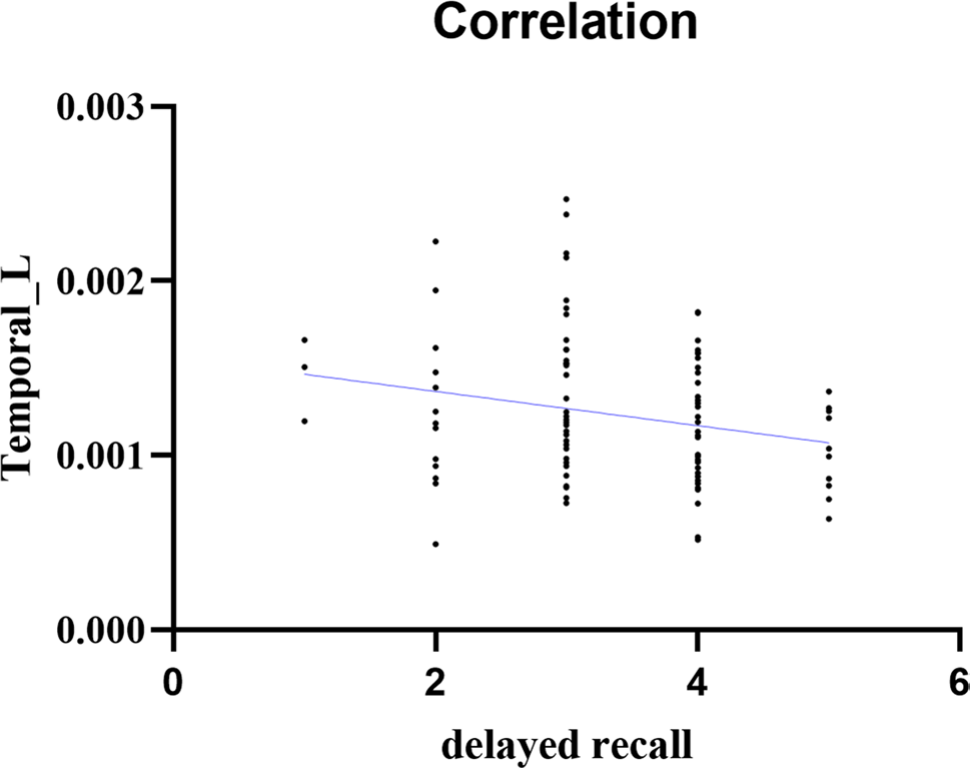
Correlation of left temporal gyrus with impaired delayed recall in patients with lung cancer and HCs

To examine the correlation between increased brain areas of BBB leakage and cognitive function, we analyzed the correlation between K^trans^ value and cognitive function of participants, and controlled age and gender factors. It was found that the K^trans^ of the left temporal gyrus was negatively correlated with delayed recall (r = -0.201, P = 0.042). We further correlated the maximum tumor diameter, serum markers (CEA, NSE, CYFRA21-1, SCC) and brain areas with increased K^trans^ in LCs and controlled age and gender factors, and we did not find any correlation between them. We also examined the relationship between leakage increased brain areas and tumor size and serum tumor markers and controlled age and gender factors, and we also found no correlation between them (Table 2, Fig. 6).

**Table 2:** Partial correlation between BBB leakage and cognitive function in patients with LC

BBB leakage	cognitive function	R	P
Temporal_L	Delayed recall	-0.201	0.042

## Discussion

This study was the first to quantitatively image BBB leakage in lung cancer patients who had not yet developed brain metastases and found that aLC exhibited higher levels of BBB leakage in some brain regions and correlated with cognitive impairment in patients. These findings provide a critical step in determining the role of BBBD in the pathogenesis of brain metastases from lung cancer and highlight the BBB as a potential diagnostic and predictive target.

BMs cause severe, uncontrollable symptoms and reduce quality of life, such as paralysis, increased intracranial pressure, or seizures, and the incidence of BM has shown an upward trend in the last decade, but there has been little progress in treatment options and poor outcomes(Soffietti et al., 2017). Therefore, it is important to be able to detect early BM in patients with lung cancer who are more prone to develop BM and to provide enhanced adjuvant therapy for them. In this study, BBB leakage examination was performed in patients who had not yet developed visible brain metastases, and increased leakage was found in patients with progressive disease. This suggests an increased risk of brain metastasis.

The development of BM involves a series of interrelated steps, starting with the invasion of cancer cells into the intravascular and/or lymphatic system. Due to the lack of a lymphatic system in the central nervous system (CNS), the only possible way for cancer cells to reach the brain is through blood circulation. The circulating tumor cells (CTCs) may circulate into the brain's microcirculation and adapt to the brain's microenvironment, resulting in the formation of micro-metastases, which eventually form visible tumors through "metastatic colonization"(Hanahan & Weinberg, 2011; Paduch, 2016). However, metastatic cells that invade the CNS parenchyma must pass through the BBB. During this process, changes occur in the cerebral vascular endothelium(Bart et al., 2000), including impaired tight junction structures and increased perivascular gaps(Liebner et al., 2000). In addition, windows corresponding to the surrounding vascular system can be found in these vessels, and the number and activity of cytosolic vesicles are increased(Shibata, 1989). Therefore, these vessels may reflect those of the tumor tissue rather than those of the CNS endothelium. As a result of these structural alterations leading to increased BBB leakiness, plasma leaks into the extracellular space outside the vasculature(Nduom et al., 2013). And our findings coincide with this process.

DCE-MRI allows the leakage of the extracellular gap within each voxel to be assessed by pharmacokinetic parameters (K^trans^), detecting leakage of the BBB in response to the destruction of the BBB. K^trans^ is defined as the volume transfer constant between the plasma and the extracellular space outside the blood vessel, and it is often used as a synonym for permeability(Tofts & Kermode, 1991). DCE-MRI has been widely used in neuro-oncology imaging(Bar-Klein et al., 2017; Kamintsky et al., 2020; Lublinsky et al., 2019; Rüber et al., 2018; Serlin et al., 2019). However, measuring leakage from a relatively intact BBB is not common. And the leakage of BBB we need to measure may be microleakage, so the patlak model is used to calculate K^trans^ because it is more accurate in measuring microleakage(Barnes et al., 2016; Heye et al., 2016; Patlak et al., 1983).

Our findings confirm that, compared to the HCs, LCs have higher levels of BBB leakage in bilateral temporal gyrus and whole brain gyrus, especially more pronounced in aLCs. It suggests that as LC progress, higher leakage of the BBB may occur in brain areas and the brain BBB integrity suffers disruption. And these changes indicate changes in the cerebral vascular endothelium in these brain regions, damage to tight junction structures and increased perivascular gaps(Soffietti et al., 2017). This signals the possibility of micro-metastasis, or a shift from BBB to BTB, as BTB is often considered “leakier” than BBB. Therefore, without further clinical intervention, visible BM may develop. Early detection of increased BBB leakage and enhanced clinical intervention are important to prevent the development of BMs.

Cognitive impairment in cancer patients has been of concern and increases the risk of developing dementia(Heck et al., 2008; Lovelace et al., 2019). Functional impairment of visuospatial/executive and delayed recall was found in patients with advanced lung cancer, and the decrease was more obvious with the progression of the disease. It is suggested that the development of lung cancer will impair cognitive function, giving priority to visuospatial/executive and delayed recall, but the specific mechanism is unclear. Some neuroimaging studies have shown that the structural and functional changes of frontal gyrus, temporal gyrus, parietal gyrus and hippocampus are related to cognitive impairment(Correa et al., 2017; de Ruiter et al., 2012; Inagaki et al., 2007; Mentzelopoulos et al., 2021; Shiroishi et al., 2017; Simó et al., 2015). Our study found that the BBB leakage in left temporal gyrus was increased and negatively correlated with delayed recall. It is well known that temporal lobe is associated with verbal and episodic memory(Buckner & Wheeler, 2001; McDermott et al., 2000; Pievani et al., 2011). Therefore, we speculate that BBB damage may be caused by the secretion of cytokines from lung cancer. Because it contains a complex network of tissue stroma, infiltrating immune cells and tumor cells, all of these cells can produce cytokines(Seruga et al., 2008). Some clinical studies have shown that peripheral cytokines are associated with cognitive decline(Andreotti et al., 2015; Ma et al., 2016; Wu et al., 2016).So, in the treatment of lung cancer, it is necessary to consider the cognitive impairment of patients, avoid inappropriate treatment, aggravate the cognitive impairment of patients, and reduce the quality of life.

The advantages of this study include no recruitment bias, and the time between clinical evaluation and neuroimaging regimens is close, usually, after clinical evaluation, an MRI examination is completed within 1 day. However, the sample size of our cohort is small, and our imaging scheme requires a gadolinium-based contrast agent, which may cause potential damage to the kidney. BBB imaging without contrast agents may have more extensive applicability.

## Conclusion

This study confirmed for the first time that there is an increase in BBB permeability in patients with non-brain metastasis before treatment, which is related to cognitive impairment, indicating that the occurrence and development of lung cancer itself can cause cognitive impairment, which is not related to treatment. In the treatment of lung cancer, the cognitive dysfunction should be fully considered to avoid aggravating the damage and reducing the quality of life. Therefore, this study provides new preliminary evidence for individualized treatment of lung cancer.

## Supplementary Information

Below is the link to the electronic supplementary material.

Supplementary file1 (DOCX 25 KB)

## Data Availability

The data from this article cannot be shared publicly due to the privacy of the individuals that participated in the study. The data will be shared on reasonable request to the corresponding author.

## Abbreviations

LC: Lung cancer
HC: Healthy control
BBB: Blood-brain barrier
BBBD: BBB dysfunction
BMs: Brain metastases
eLC: Early lung cancers
aLC: Advanced lung cancers
CNS: Central nervous system
DCE-MRI: Dynamic enhanced magnetic resonance imaging
ROI: Region of interest
VIF: Vascular input function
AAL: Anatomical labeling
CEA: Carcinoembryonic antigen
NSE: Neuron-specific enolase
CYFRA21-1: Cytokeratins21-1
SCC: Squamous cell carcinoma antigen
Temporal_L: Left temporal gyrus
Temporal_R: Right temporal gyrus
Gyrus: The whole brain gyrus matter
