# Supplementary material for: Cognitive changes are associated with increased blood-brain barrier leakage in non-brain metastases lung cancer patients
Source: Brain Imaging Behav. 2022 Nov 22;17(1):90–9. doi: 10.1007/s11682-022-00745-3 (PMC9922230; doi:10.1007/s11682-022-00745-3)
Supplement: Supplementary file 1 — Supplementary file1 (DOCX 25 KB) [file 11682_2022_745_MOESM1_ESM.docx]

Supplementary Materials

Table S1 Comparison of BBB leakage between patients with lung cancer and HCs.

|  | HCs(*n*=29) | | LCs(*n*=75) | *H* | *P* |
| --- | --- | --- | --- | --- | --- |
| Temporal_L | 9.71E-04 (7.34E-04, 1.32E-03) | 1.26E-03 (1.04E-03, 1.51E-03) | | -2.852 | 0.004 |
| Temporal_R | 1.21E-03 (9.67E-04, 1.61E-03) | 1.46E-03 (1.25E-03, 1.78E-03) | | -2.794 | 0.005 |
| Gray | 6.26E-04 (5.47E-04, 7.85E-04) | 7.09E-04 (6.35E-04, 8.22E-04) | | -2.055 | 0.040 |

Data are expressed as Quartile. The P values are obtained by using Kruskal-Wallis Test. Temporal_L*, left temporal gyrus. Temporal_R, right temporal gyrus.* Gray*, the whole brain gray matter.*

Table S2 Comparison of BBB leakage between patients with lung cancer at different stages and HCs.

|  | HCs(N=29) | eLCs(N=39) | aLCs(N=36) | H | P |
| --- | --- | --- | --- | --- | --- |
| Temporal_L | 9.71E-04 (7.34E-04, 1.32E-03) | 1.22E-03 (1.03E-03, 1.50E-03) | 1.33E-03 (1.07E-03, 1.53E-03) | 8.901 | 0.012 |
| Temporal_R | 1.21E-03 (9.67E-04, 1.61E-03) | 1.44E-03 (1.16E-03, 1.76E-03) | 1.46E-03 (1.31E-03, 1.86E-03) | 8.480 | 0.014 |
| Gray | 6.26E-04 (5.47E-04, 7.85E-04) | 6.83E-04 (5.72E-04, 7.60E-04) | 7.35E-04 (6.68E-04, 8.39E-04) |  |  |

Comparison of BBB leakage between patients with lung cancer at different stages and HCs. Data are expressed as Quartile. The P values are obtained by using Kruskal-Wallis Test. Temporal_L*, left temporal gyrus. Temporal_R, right temporal gyrus.* Gray*, the whole brain gray matter.*

Table S3-5. Multiple comparisons of the group. Asymptotic significances (2-sided tests) are displayed. The significance level is 0.05.

^a^. Significance values have been adjusted by the Bonferroni correction for multiple tests. 1= HCs; 2= eLCs; 3= aLCs.

Table S3

| **Temporal_L** | | | | | |
| --- | --- | --- | --- | --- | --- |
| Sample 1-Sample 2 | Test Statistic | Std. Error | Std. Test Statistic | Sig. | Adj. Sig.^a^ |
| 1-2 | -15.889 | 7.397 | -2.148 | 0.032 | 0.095 |
| 1-3 | -21.986 | 7.527 | -2.921 | 0.003 | 0.010 |
| 2-3 | -6.096 | 6.972 | -0.874 | 0.382 | 1.000 |

Table S4

| **Temporal_R** | | | | | |
| --- | --- | --- | --- | --- | --- |
|  | Test Statistic | Std. Error | Std. Test Statistic | Sig. | Adj. Sig.^a^ |
| 1-2 | -15.691 | 7.397 | -2.121 | 0.034 | 0.102 |
| 1-3 | -21.404 | 7.527 | -2.844 | 0.004 | 0.013 |
| 2-3 | -5.714 | 6.972 | -0.819 | 0.413 | 1.000 |

Table S5

| **ktransGray** | | | | | |
| --- | --- | --- | --- | --- | --- |
|  | Test Statistic | Std. Error | Std. Test Statistic | Sig. | Adj. Sig.^a^ |
| 1-2 | -5.789 | 7.397 | -0.783 | 0.434 | 1.000 |
| 1-3 | -21.970 | 7.527 | -2.919 | 0.004 | 0.011 |
| 2-3 | -16.182 | 6.972 | -2.321 | 0.020 | 0.061 |

Table S6. Correlation between BBB leakage and cognitive function in patients with LC

| BBB leakage | cognitive function | R | P |
| --- | --- | --- | --- |
| Temporal_L | Delayed recall | -0.201 | 0.042 |

The P values are obtained by using spearman correlation analysis, The correlation is significant at the 0.05 level.
